# The significance of an immunohistochemical marker-based panel in assisting the diagnosis of parathyroid carcinoma

**DOI:** 10.1007/s12020-024-03687-6

**Published:** 2024-02-10

**Authors:** Ya Hu, Shengwei Mo, Jinheng Xiao, Ming Cui, Qingyuan Zheng, Tianqi Chen, Xiaoyan Chang, Quan Liao

**Affiliations:** 1 Department of General Surgery, State Key Laboratory of Complex Severe and Rare Diseases, Peking Union Medical College Hospital, Chinese Academy of Medical Science and Peking Union Medical College, Beijing, China; 2 Department of Pathology, Peking Union Medical College Hospital, Chinese Academy of Medical Sciences and Peking Union Medical College, Beijing, China; 3 Medical Research Center, Peking Union Medical College Hospital, Chinese Academy of Medical Sciences and Peking Union Medical College, Beijing, China

**Keywords:** Parathyroid carcinoma, Immunohistochemistry, Differential diagnosis, Parafibromin, Ki-67, E-cadherin

## Abstract

**Purpose:**

Parathyroid carcinoma (PC) is an endocrine malignancy with a poor prognosis. However, the diagnosis of PC is still a difficult problem. A model with immunohistochemical (IHC) staining of 5 biomarkers has been reported from limited samples for the differential diagnosis of PC. In the present study, a series of IHC markers was applied in relatively large samples to optimize the diagnostic model for PC.

**Methods:**

In this study, 44 patients with PC, 6 patients with atypical parathyroid tumors and 57 patients with parathyroid adenomas were included. IHC staining for parafibromin, Ki-67, galectin-3, protein-encoding gene product 9.5 (PGP9.5), E-cadherin, and enhancer of zeste homolog 2 (EZH2) was performed on formalin-fixed, paraffin-embedded tissue samples. The effects of clinical characteristics, surgical procedure, and IHC staining results of tumor tissues on the diagnosis and prognosis of PC were evaluated retrospectively.

**Results:**

A logistic regression model with IHC results of parafibromin, Ki-67, and E-cadherin was created to differentiate PC with an area under the curve of 0.843. Cox proportional hazards analysis showed that negative parafibromin staining (hazard ratio: 3.26, 95% confidence interval: 1.28–8.34, *P* = 0.013) was related to the recurrence of PC.

**Conclusion:**

An IHC panel of parafibromin, Ki-67 and E-cadherin may help to distinguish PC from parathyroid neoplasms. Among the 6 IHC markers and clinical features examined, the risk factor related to PC recurrence was parafibromin staining loss.

## Introduction

Primary hyperparathyroidism (PHPT) is a common endocrine disorder. The proliferative parathyroid gland autonomously releases excessive parathyroid hormone (PTH), which causes hypercalcemia and related complications, such as osteoporosis, bone fracture, urinary calculi, renal failure, and neuropsychiatric symptoms. According to the histopathological evaluation, PHPT can be caused by parathyroid adenoma (PA) and parathyroid carcinoma (PC). PC accounts for only approximately 1% of PHPT cases in Western countries, while more than 5% of patients with PHPT suffer from malignancy in China and Italy [1, 2]. In recent years, the incidence of PC has increased substantially in many countries [3–5]. Although the 10-year overall survival rate is reported to be 49–91%, the outcome of PC is still poor [6–8]. Approximately 50% of patients with PC suffer from frequent recurrence or metastasis, and multiple resections are needed [9]. The leading cause of patient mortality from PC is unmanageable hypercalcemia rather than tumor invasion. Currently, the efficacy of chemotherapy and radiotherapy in PC is frustrating. Although a few case reports about molecular targeted therapy and immunotherapy showed a glimmer of hope, the management of PC largely depends on surgery. Radical surgery, including en bloc resection of parathyroid tumors, the ipsilateral thyroid lobe, surrounding involved tissue and the ipsilateral central cervical compartment of lymph nodes, is suggested to decrease the risk of recurrence and metastasis [6].

The differential diagnosis of PC from benign parathyroid neoplasm is difficult before or during operation due to their considerable overlap in clinical presentation and biochemical analysis results. This dilemma might be more prominent in China, where diagnosis delay results in serious clinical symptoms and large lesions that may mimic parathyroid malignancy. For histopathological evaluation of tumor samples after the operation, the World Health Organization (WHO) classification criteria describe the typical presentations of PC, including definite tumor invasion into adjacent tissue, tumor invasion in blood vessels or perineural space located at capsular or extracapsular tissues, or metastasis [10]. However, a subset of borderline parathyroid tumors presenting with worrisome features, such as prominent adherence to surrounding structures, pronounced fibrosis bands and thick capsules, trabecular growth, nuclear atypia, and high mitotic rates, should not be recognized as PCs according to previous criteria [11, 12]. Furthermore, bias in the differential diagnosis of PC cannot be avoided based merely on morphological characteristics under a microscope. Several biomarkers have been introduced to resolve this dilemma. Parafibromin, encoded by *CDC73*, is used as the first and most important biomarker in the differential diagnosis of PC [13, 14]. Moreover, other biomarkers, such as Rb1, galectin-3, PGP9.5, and Ki-67, have also been applied [15, 16]. In 2019, a study conducted by Silva-Figueroa et al. [17] introduced a nomogram based on immunohistochemical (IHC) staining of parafibromin, Rb, Ki-67, E-cadherin, PGP9.5, and galectin-3 to assist in the diagnosis of PC. However, a predictive model with 5 biomarkers seems slightly cumbersome in clinical practice. The importance of these biomarkers in the recurrence and survival of patients with PC is still less known.

Therefore, we performed IHC staining of a series of biomarkers, including parafibromin, Ki-67, PGP9.5, E-cadherin, galectin-3 and EZH2, in a cohort of 44 patients with PC. Another 63 patients with atypical parathyroid tumor (APT) and typical PA were used as controls. This study was designed to reevaluate the diagnostic power of these biomarkers in this Chinese cohort and to assess the impact of IHC staining results, clinical features, and surgical extent on the clinical outcome of these patients.

## Materials and methods

### Patient cohort and data collection

A total of 107 patients were included in this study, including 44 with PC, 6 with APT, and 57 with PA. All patients underwent surgical procedures at the Peking Union Medical College Hospital, a tertiary university hospital. Some of the cases were included in our previous study [9]. No history of neck radiation was identified in these patients. One patient with PC harbored a germline *CDC73* mutation, and one patient with PA was identified with a germline *MEN1* mutation. Clinical information, including sex, age at diagnosis, initial surgical procedure, and pathological evaluation results, was collected retrospectively from medical records. Relapse and survival status were acquired through outpatient records and regular follow-up. The diagnosis of parathyroid neoplasm was based on the WHO 2022 criteria [10]. The diagnosis of PC was based on (1) infiltration of the tumor tissue through the capsule into adjacent structures; (2) tumor infiltration to extracapsular vascular or perineural areas; and (3) metastasis in the lymph node or distal location. APT is defined in the WHO 2022 criteria as a parathyroid neoplasm presenting with atypical cytological and architectural features, while mention of unequivocal evidence of malignancy above was absent. These worrisome features include cellular nests in the connective tissue septum, considerable fibrosis within the tissue, tumor cells found in the tumor capsule or suspected invasion, and cytologic atypia such as mitotic activity >5/10 mm^2^ or Ki-67 index >5%. All participants provided written informed consent, and the Institutional Ethics Review Board of Peking Union Medical College Hospital approved this study (S-K636).

### Immunohistochemical staining

**Fig. 1:**
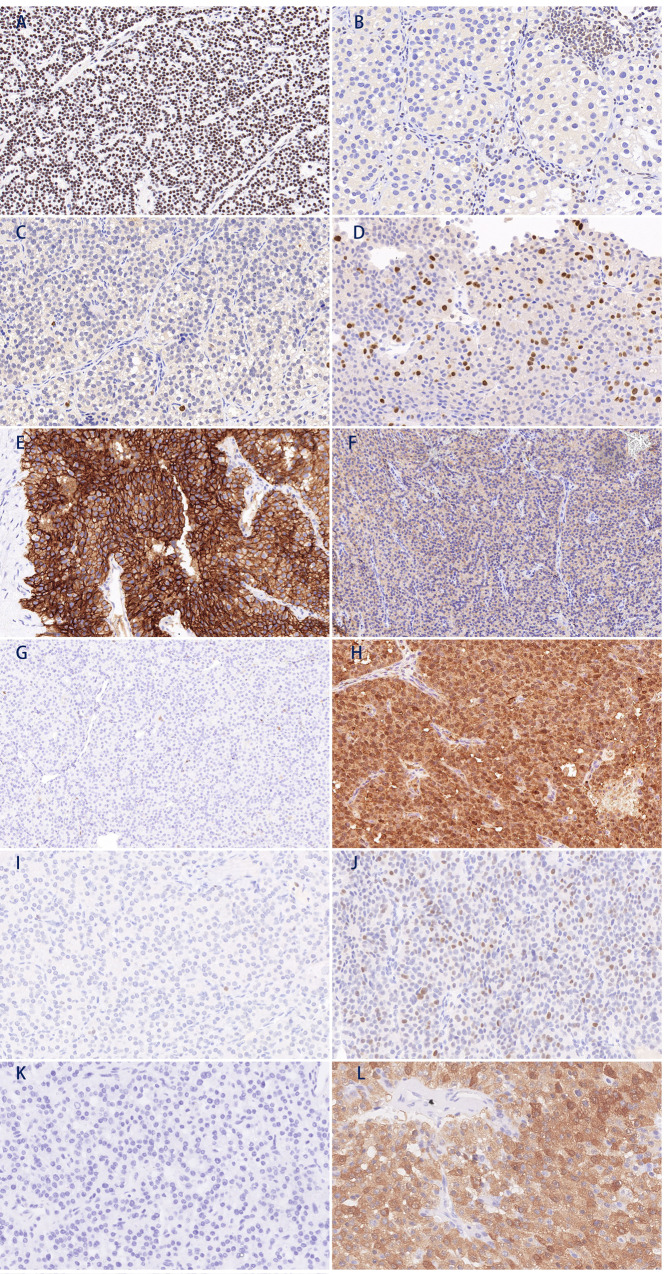
Representative immunostaining micrographs of parathyroid carcinoma tissues **A** normal parafibromin staining; **B** parafibromin staining loss; **C** low Ki-67 index; **D** high Ki-67 index; **E** normal E-cadherin positive staining; **F** E-cadherin staining loss; **G** normal galectin-3 negative staining; **H** positive galectin-3 staining; **I** normal EZH2 negative staining; **J** positive EZH2 staining; **K** normal PGP9.5 negative staining; **L** positive PGP9.5 staining

Immunohistochemical staining for parafibromin, Ki-67, PGP9.5, galectin-3, E-cadherin, and EZH2 was performed on formalin-fixed, paraffin-embedded tissue samples. These PC tissue sections originated from 21 primary PC tissues, 20 recurrent tumor tissues, and 3 pulmonary metastatic lesions. A homemade 2 mm diameter tissue array from 6 APT and 57 PA continuously collected between May 2018 and March 2019 was used as a control, in which the maximum tumor diameter exceeded 1 centimeters. All samples of the control group, including PA and APT, were collected from primary foci. After deparaffinization and hydration, tissue sections were subjected to heat-induced antigenic retrieval with antigen-retrieval solution containing ethylenediaminetetraacetic acid or citric acid. After incubation in a 3% H_2_O_2_ solution to block endogenous peroxidase, 3% bovine serum albumin was used to block nonspecific antibody binding sites. Then, the slides were incubated with primary antibodies for different proteins overnight at 4 °C (Table 1). Horseradish peroxidase-labeled secondary antibodies and diaminobenzidine were used according to the manufacturer’s instructions. Finally, the slides were counterstained with hematoxylin. The stained sections were independently evaluated by two investigators who were blinded to the related information. Negative parafibromin staining was defined as complete loss of nuclear expression in tumor cells with retained positive staining being observed in internal positive controls (i.e., vascular endothelial and stromal cells) (Fig. 1). The entire tissue section was examined to evaluate the proportion of staining for each biomarker. The interpretation of immunohistochemical staining results was in accordance with the literature and our previous study [17].

**Table 1:** The antibodies and judging criteria in the immunohistochemical staining in this study

Biomarker	Antibody source	Catalog no.	Dilution	Cellular location	Abnormality	Staining thresholds for abnormality	Antigen-retrieval solution
Parafibromin	Abcam	ab223840	1:200	Nucleus	Negative	Total negative	EDTA
Ki-67	Servicebio	GB111499	1:800	Nucleus	Positive	>5%	EDTA
PGP9.5	ZETA	ZM160	1:500	Cytoplasm	Positive	>50%	EDTA
Galectin-3	Origene	UMAB157	1:200	Cytoplasm	Positive	>30%	EDTA
E-cadherin	Leica	36B5	1:1	Membrane/Cytoplasm	Negative	<90%	EDTA
EZH2	Abcam	ab283270	1:100	Nucleus	Positive	>5%	Citric acid

*EDTA* Ethylenediaminetetraacetic acid

### Statistical analysis

Continuous variables are presented as the mean ± standard deviation (SD), and discrete data are described as numbers or corresponding percentages. T tests, Chi-square tests or Fisher’s exact tests were used to compare variables between groups. Disease-free survival (DFS) was defined as the interval from the date of initial surgery to first recurrence, metastasis, or last follow-up for PC. Subjects with distant metastasis before the initial operation or without remission after surgery were excluded from the calculation of DFS. In addition, 95% confidence intervals (CIs) were calculated for hazard ratios (HRs). SPSS for Windows (version 21.0, SPSS Inc., Chicago, IL, USA) was used for statistical calculation. *P* values < 0.05 were considered statistically significant.

## Results

### The clinical characteristics of the subjects

Among 107 samples, 44 PC patients were included in this retrospective study. The average age of the PC patients was 42.7 ± 14.3 years, which was lower than that in the control group (54.1 ± 11.7 years, *P* < 0.001) (Table 2). Higher serum intact PTH (iPTH) levels and larger tumors were found in the PC group. The median follow-up time was 61.5 months (range: 3–323 months) for PC. None of the patients with APT or PA experienced tumor persistence or recurrence in the present cohort, with a median follow-up of 34.2 months.

**Table 2:** The difference of clinical features and immunohistochemical staining results between parathyroid carcinoma group and control group

Factors	PC group	Control group	*P* value
Cases number	44	63	
Age at diagnosis (years)	42.7 ± 14.3	54.1 ± 11.7	<0.001*
Male vs female	18:26	19:44	0.250
Serum iPTH (pg/ml)^a^	1383.5 ± 811.7	411.3 ± 545.1	<0.001*
Serum calcium (mmol/L)	3.18 ± 1.15	2.95 ± 0.41	0.209
Maximum tumor diameter (cm)^a^	3.3 ± 1.0	2.4 ± 1.0	<0.001*
Parafibromin loss	24/44 (54.5%)	2/63 (3.2%)	<0.001*
Ki-67 index > 5%	20/44 (45.5%)	9/63 (14.3%)	<0.001*
E-cadherin staining loss^a^	21/44 (47.7%)	11/59 (18.6%)	0.002*
Positive Galectin-3 staining^a^	27/44 (61.4%)	4/62 (6.5%)	<0.001*
Positive PGP9.5 staining^a^	17/44 (38.6%)	7/61 (11.5%)	0.001*
Positive EZH2 staining^a^	14/44 (31.8%)	0/61 (0)	<0.001*

Parathyroid adenoma and atypical parathyroid tumor were included in control group

*PC* parathyroid carcinoma, *iPTH* intact parathyroid hormone

*P < 0.05

^a^Data of a few cases were unavailable for this variable.

### Correlation of immunohistochemical staining of markers and clinical features

**Fig. 2:**
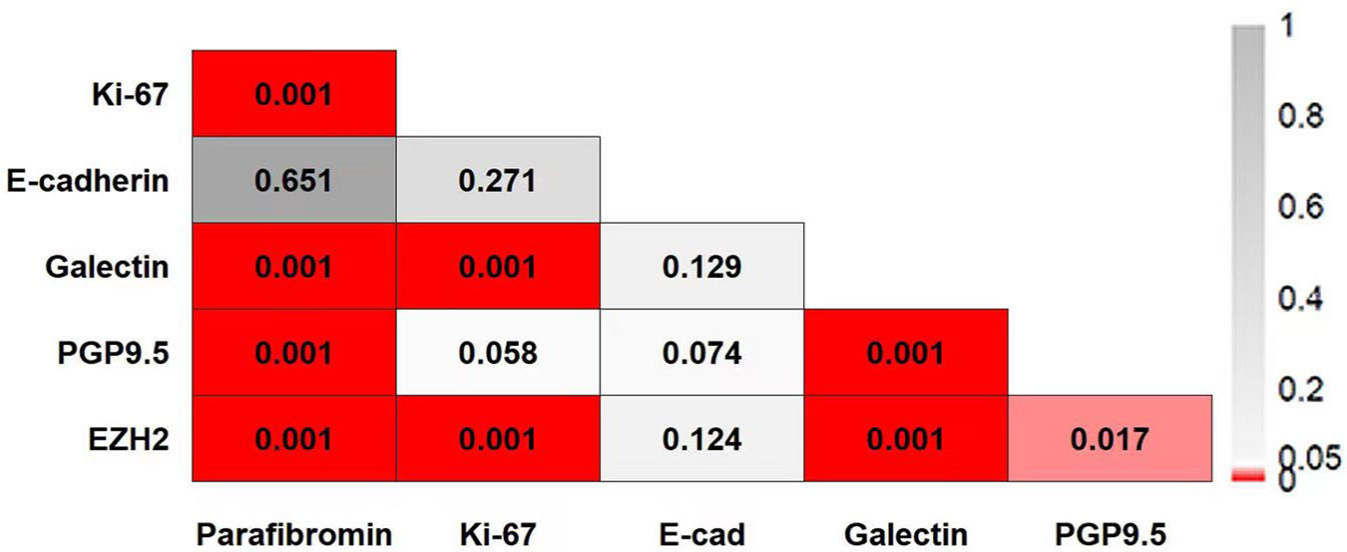
The correlation matrix among the expression of 6 immunohistochemical biomarkers in 107 patients with parathyroid neoplasms (PC, APT and PA). The *P* value of each statistical analysis is shaded with a different color; negative parafibromin expression in parathyroid neoplasms was significantly associated with overexpression of Ki-67, galectin-3, and PGP9.5

For the patients with parathyroid neoplasms (PC, APT and PA), higher serum iPTH was associated with Ki-67 > 5% (*P* = 0.035), positive EZH2 (*P* < 0.001), galectin-3 (*P* = 0.002) and PGP9.5 staining (*P* = 0.027). Lower age was also found in patients with negative parafibromin staining (*P* < 0.001) and positive EZH2 (*P* = 0.013), E-cadherin (*P* = 0.039), galectin (*P* < 0.001) and PGP9.5 staining (*P* = 0.001). No difference in the expression of all 6 markers was found between male or female patients and patients with or without cystic tumors. Among 107 patients in this cohort, negative parafibromin expression in parathyroid neoplasms was significantly associated with overexpression of Ki-67, galectin-3, and PGP9.5, while E-cadherin staining loss was independent of other biomarkers (Fig. 2).

### The value of the IHC panel in the differential diagnosis of PC

In IHC, higher percentages of parafibromin loss, Ki-67 > 5% and positive galectin expression, E-cadherin loss and positive PGP9.5 expression were detected in the PC group than in the control group (Table 2). By multivariable logistic regression, a model including parafibromin (*P* < 0.001), Ki-67 (*P* = 0.033) and E-cadherin (*P* < 0.001) was established to differentiate PC from other parathyroid neoplasms. The AUC (area under the curve) of the receiver operating characteristic curve for this combination model was 0.865 (95% CI 0.788–0.942), which was higher than that of the predictive model including only parafibromin staining (AUC = 0.757, 95% CI 0.656–0.858).

### The correlations of immunohistochemical markers and clinical outcomes in patients with PC

Among 44 patients with PC, univariate Cox regression analysis showed that parafibromin loss was a potential possible risk factor for tumor relapse or metastasis (HR: 3.26, 95% CI: 1.28–8.34, *P* = 0.013), while none of these markers was found to be related to mortality of PC (Table 3). In multivariable Cox regression analysis including IHC markers and clinical features, including sex, age, and surgical extent, only parafibromin loss was identified to be associated with DFS (HR: 5.805, 95% CI 1.02–33.04, *P* = 0.047). However, neither IHC markers nor clinical features were found to be associated with OS.

**Table 3:** Risk factors for the disease-free survival and overall survival of patients with parathyroid carcinoma in univariate Cox proportional hazard analysis

Factors	Disease-free survival			Overall survival		
	HR	95% CI	*P* value	HR	95% CI	*P* value
Age at diagnosis (years)	1.01	0.98–1.05	0.503	1.03	0.97–1.10	0.315
Male vs female	1.02	0.45–2.30	0.968	1.25	0.27–5.69	0.777
Cystic tumors (*n* = 41)	0.86	0.28–2.59	0.786	2.15	0.40–11.51	0.371
Maximum diameter of initial tumor (cm) (*n* = 39)	0.76	0.43–1.34	0.349	0.52	0.16–1.71	0.279
Serum iPTH at diagnosis (pg/ml) (*n* = 39)	1.00	1.00–1.00	0.179	1.00	1.00–1.00	0.403
Serum calcium level at diagnosis (mmol/L)	1.19	0.82–1.72	0.360	0.88	0.54–1.46	0.626
En-bloc vs local resection	0.95	0.38–2.37	0.906	1.29	0.23–7.15	0.769
Parafibromin loss	3.26	1.28–8.34	0.013*	0.89	0. 20–4.04	0.882
Ki-67 index >5%	2.18	0.93–5.14	0.075	0.71	0.16–3.17	0.652
E-cadherin staining loss	0.61	0.26–1.39	0.237	0.56	0.12–2.56	0.453
Positive galectin staining	1.68	0.65–4.32	0.281	1.04	0.20–5.39	0.959
Positive PGP9.5 staining	1.58	0.69–3.62	0.275	0.78	0.17–3.48	0.742
Positive EZH2 staining	1.47	0.63–3.40	0.373	0.90	0.17–4.66	0.895

*CI* confidence interval, *HR* hazard ratio, *iPTH* intact parathyroid hormone

*P < 0.05

## Discussion

Even though a relatively long survival is common for most patients with PC, the quality of life is seriously damaged by repeated recurrence and multiple metastases. Moreover, the differential diagnosis of PC is still challenging. A series of biomarkers has been introduced to increase the diagnostic accuracy. A previous study by Silva-Figueroa et al. created a diagnostic nomogram with 5 IHC biomarkers for differentiating PC from PA in a cohort including 21 PCs. In the present study, we further optimized a diagnostic model with the IHC results of parafibromin, Ki-67, and E-cadherin from 44 PC tissue samples. We also found that no other biomarker except parafibromin was related to the outcome of patients with PC.

Inactivated mutation of the *CDC73* gene results in loss of parafibromin staining in the nuclei of tumor cells [13]. Parafibromin is the first identified biomarker for PC, with an estimated sensitivity of 68% and specificity of 100% [14]. Parafibromin staining loss was also associated with poor outcomes of PC in studies by other investigators and our previous meta-analysis [18, 19]. In another of our previous studies, the DFS of PC patients was influenced mainly by parafibromin staining loss in the tumor cell nucleus rather than resection extent in the initial surgery [9]. Therefore, parafibromin staining plays a critical role in the panel for differential diagnosis [17]. In our present study, parafibromin staining loss was not only an important marker in the differential diagnosis of PC but also the only prognostic factor for PC among a group of markers. *CDC73* abnormalities may be a key target point for molecular therapy.

In our previous study, a high Ki-67 index in tumors was a risk factor associated with PC relapse [20]. This was consistent with studies by other authors [17, 21, 22]. In our present study, we reaffirmed that a Ki-67 index >5% was a marker associated with recurrence or metastasis, while its correlation with DSF did not reach statistical significance in survival analysis. E-cadherin is a calcium-dependent transmembrane protein encoded by the *CDH1* gene that plays an important role in maintaining intercellular adhesion and the phenotype of epithelial tissue. Loss of E-cadherin is related to enhanced cell motility and decreased contact inhibition, resulting in invasion and metastasis of epithelial cancer cells [23]. Membranous E-cadherin staining loss was found in most PCs [24, 25]. In our present study, we built a predictive model based on staining of parafibromin, Ki-67 and E-cadherin for the differential diagnosis of PC with an AUC of 0.865, which is close to that from the study by Silva-Figueroa et al. [17]. However, the difference between these 2 studies is that a combination of 3 instead of 5 biomarkers was used in our study, reducing operational complexity in clinical work.

The other 3 IHC markers were not included in our present predictive model, even though their expression levels were different between the PC and control groups. PGP9.5 is encoded by the *UCHL1* (ubiquitin carboxyl-terminal esterase L1) gene, which regulates the cell cycle. It was suggested as a supplementary marker for PC diagnosis in previous studies [17, 26, 27]. This was reconfirmed in our present study. In our previous study comparing expression levels using a gene expression profile chip, the expression level of *UCHL1* in PC was significantly higher than that in PA, with a fold change of 26.5 [28]. In addition, PGP9.5 was also a significant predictor for relapse after initial surgery [19]. Galectin-3 is a β-galactoside-binding lectin involved in multiple cellular activities, including cell proliferation, apoptosis, inflammation, and migration in various tumors, heart disease, autoimmune disease, and liver fibrosis [29, 30]. It was used as a marker for the differential diagnosis of PC from benign tumors in several previous studies [26, 31, 32]. *EZH2* was found to be a critical gene in the development of the parathyroid in a mouse model [33]. It was proposed that *EZH2* overexpression was involved in important pathways of parathyroid tumor development [34]. The expression of *EZH2* in PC tissue was found to be higher than that in PA [35]. It was also identified as a candidate for the differential diagnosis of PC in our previous study of the expression profile, which revealed that a higher level of *EZH2* mRNA occurred in PC than in PA [28]. Here, IHC staining of these 2 biomarkers was performed further to identify the difference in protein expression level in different subsets of parathyroid neoplasms, while the prognostic effect of PGP9.5, Galectin-3 and EZH2 was not identified in our present study. Due to the close correlation with parafibromin staining loss, these 3 biomarkers were not included in our present IHC model in logistic regression.

Several shortcomings of this study should be mentioned here. First, the PC sample size was still limited due to low incidence, even though our present study included one of the largest single-center cohorts in recent years. Insufficient sample size may lead to underestimation of the effect of certain IHC markers in diagnosis and predicting prognoses. Second, some of the samples in our study were from metastatic or recurrent PC lesions, and their IHC results may be different from those from primary lesions, even though our previous studies did not find different IHC expression patterns of parafibromin in primary and secondary foci. Bias could not be avoided in this retrospective study, and prospective observation with a larger cohort is underway to validate our findings.

## Conclusion

Differential diagnosis of PC from benign parathyroid neoplasm remains difficult with histopathological features. An IHC panel of parafibromin, Ki-67 and E-cadherin may help to resolve this dilemma. Among the 6 IHC markers and clinical features, the risk factor related to recurrence was parafibromin staining loss.

## Data Availability

The datasets generated during and/or analyzed during the current study are available from the corresponding author upon reasonable request.
